# QL-HIT2F: A Q-Learning-Aided Adaptive Fuzzy Path Planning Algorithm with Enhanced Obstacle Avoidance

**DOI:** 10.3390/s26010200

**Published:** 2025-12-27

**Authors:** Nana Zhou, Fengjun Zhou, Changming Li, Jianning Zhong

**Affiliations:** 1 School of Computer Science, Shandong Xiehe University, Jinan 250109, China; zhoufengjunde@126.com; 2 College of Electrical Engineering, Sichuan University, Chengdu 610065, China; lichangming@stu.scu.edu.cn (C.L.); jianning_zhong@163.com (J.Z.)

**Keywords:** robot path planning, hierarchical type-2 fuzzy, Q-learning

## Abstract

There has been significant interest in solving robot path planning problems using fuzzy logic-based methods. Recently, the Genetic Algorithm-based Hierarchical Interval Type-2 Fuzzy (GA-HIT2F) system has been introduced as a novel planner in this domain. However, this method lacks adaptability to changes in target points, and insufficient flexibility can lead to planning failures in local minimum traps, making it difficult to apply to complex scenarios. In this paper, we identify the limitations of the original GA-HIT2F approach and propose an enhanced Q-Learning-aided Adaptive Hierarchical Interval Type-2 Fuzzy (QL-HIT2F) algorithm for path planning. The proposed planner incorporates reinforcement learning to improve a robot’s capability to avoid collisions with special obstacles. Additionally, the average obstacle orientation (AOO) is introduced to optimize the robot’s angular adjustments. Two supplementary robot parameters are integrated into the reinforcement learning action space, along with fuzzy membership parameters. The training process also introduces the concepts of meta-map and sub-training. Simulation results from a series of path planning experiments validate the feasibility and effectiveness of the proposed QL-HIT2F approach.

## 1. Introduction

Smart mobile robots (SMR) are utilized in a wide range of industries for the sake of solving complex, tedious and dangerous tasks in manifold engineering environments, improving work quality and promoting efficient output, such as precise and flexible handling of goods in manufacturing and logistics, the exploration, detection and special tasks in high-risk environments, etc. [1,2,3,4,5]. Enabling robotic systems with superior autonomous navigation is crucial to mobile robot intelligence. Safe and reliable path planning is the basis and pivot of any navigation technology. Path planning requires obtaining a path that enables the robot to reach the target and avoid the obstacles along the way. Currently, a large number of path planning algorithms have been proposed, mainly including the visual graph [6] and cell decomposition [7] based on geometric principles, the artificial potential field (APF) [8] method with the assumption of the existence of fabricated target gravity and obstacle repulsion field, A* [9] and D* [10] algorithms through discrete graph search, others like numerous heuristic methods based on nature inspired optimization algorithms, and sampling-based methods of a family of probabilistic road map (PRM) and rapidly exploration random trees (RRT) [11,12]. However, the above methods must obtain complete global information and there are uncertainties in the process of robot path planning as well, such as uncertain expressions with errors from sensors and actuators. Considering this situation of local path planning (LPP), fuzzy logic-based path planning methods [13] are an effective method that can handle uncertainty in path planning.

The advantages of fuzzy logic come from the great performance of fuzzy systems [14,15,16] to handle uncertainty and the universal approximation of nonlinear functional relations, which has led to a broad applications of fuzzy logic methods in robot path planning [17,18,19,20,21,22]. When it is applied to path planning, fuzzy logic generally models the robot’s obstacle avoidance behavior and then designs some kind of fuzzy system, while the model expressing the obstacle avoidance behavior is transformed into a suitable set of fuzzy rules by which the robot performs corresponding planning for navigation toward the target. The fuzzy system takes the relevant information it obtains (obstacle distances, etc.) as system inputs and, by fuzzy reasoning, finally outputs the necessary variables for path planning such as steering angle and step length, etc. Additionally, fuzzy logic is commonly developed as a local path planning (LPP) method and is often employed as a complementary planning stage following global planning. For example, in [23], a set of guidance points derived from D* Lite through a proposed forward search optimization technique was utilized as sub-goals within an improved fuzzy inference system (FIS), which functions as a local planner in their hybrid framework.

In [22], a hierarchical interval type-2 fuzzy system (HIT2F) was proposed for local path planning. This approach leverages the capability of interval type-2 fuzzy inference to handle uncertainties in unknown environments, while the hierarchical structure mitigates rule explosion and simplifies system complexity. The system takes as inputs the distance between the detected obstacle and the self-mobile robot (SMR), as well as the angle formed by the robot, the obstacle, and the target point, ultimately outputting the linear and angular velocities of the robot to guide its motion in local path planning. In addition, in the design of each affiliation function, this study also applies the meta-heuristic intelligent genetic algorithm (GA) to obtain the optimal parameters of the affiliation functions as best as possible, and proposes a parameters-optimized version of the HIT2F algorithm (called GA-HIT2F) with better planning effect. However, this planner requires re-optimization of its fixed parameters whenever the map or target changes. Furthermore, its path planning may fail in particular wall-like environments that create local minimum traps.

Considering the above defects, the motivation of this research is to make up for the weakness of the GA-HIT2F and the problems we focus on are listed as follows: (1) the way to self-adjust the affiliation function parameters for an unknown environment; (2) the way to flee from local minimum in LPP for specific obstacles (irregular obstacles); (3) crucial variables to determinate the state and action space of the mobile robot. Therefore, building upon the GA-HIT2F path planner and inspired by the work in [24,25,26,27,28,29], we introduce a reinforcement learning mechanism to enable dynamic, adaptive parameter adjustment within the HIT2F framework. Additionally, two new parameters are incorporated: an AOO switch and the detected distance. Both are designed to enhance obstacle avoidance learning and performance. This paper first explains the principles of defects in the parameters of the original method and the potential failed solution in some special wall-shape maps and then illustrates the necessity of dynamic adjustment of the affiliation parameters; furthermore, we propose and implement a new planning algorithm, QL-HIT2F. The improved algorithm introduces the concept of average obstacle orientation and uses the Q-Learning method, one of the reinforcement learning methods, for dynamic adaptive adjustment of the affiliation parameters in HIT2F system. The meta-map is also proposed to train the SMR, and the effectiveness of the fully sub-trained SMR to be able to plan satisfactory obstacle avoidance paths in the generalized map environment is verified.

## 2. The Original GA-HIT2F and Its Performance Analysis

### 2.1. Basic GA-HIT2F Path Planning Algorithm

**Figure 1:**
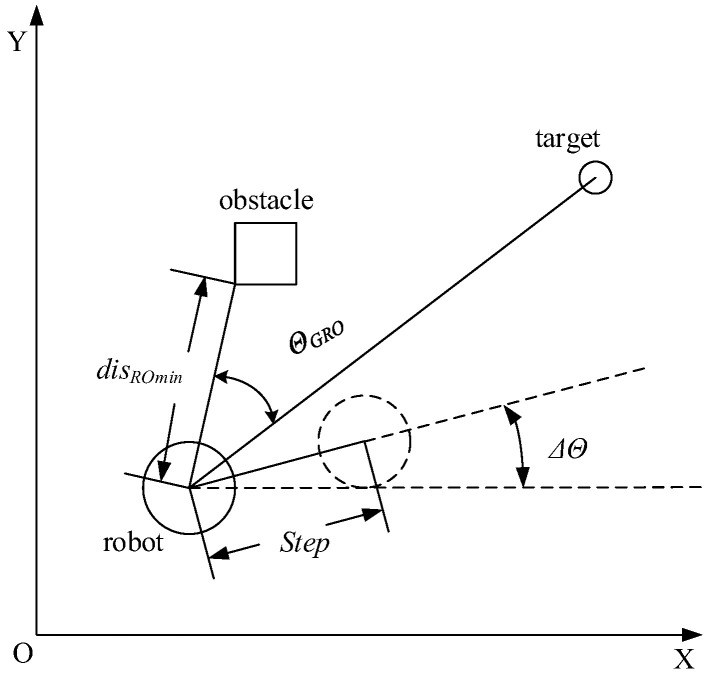
Illustration of the detection of SMR of GA-HIT2F.

As described in [22], some excellent achievements have been made; however, some problems exist. Based on the analysis of the work in [22], this paper renamed some variables and functions according to the steps and principles of the HIT2F algorithm given in [22] for simple representation, and a detailed pseudo-code version of GA-HIT2F is shown in the following flow (Algorithm 1):
**Algorithm 1** The origin GA-HIT2F1: Input necessary Map and Robot Information 2: $\left[R_{x},R_{y},\theta_{R}]\leftarrow \mathrm{InitRobotState}(\right)$; 3: Path←ϕ; 4: $Path.append([R_{x},R_{y}])$; 5: $\mathrm{While}\;dis_{RG}>dis_{thr}$ 6:  $[dis_{RO\min},\theta_{GRO}]\leftarrow \mathrm{RangeSensor}\left(RobotState\right)$; 7:  $f_{in1}\leftarrow dis_{RO\min}/(dis_{range}/5);$ 8:  $f_{in2}\leftarrow \theta_{GRO};$ 9:  $[f_{out1},f_{out2}]\leftarrow \mathrm{GAHIT}2\mathrm{Fsystem}\left(f_{in1},f_{in2}\right);$ 10:  $step\leftarrow m•f_{out1};$ 11:  $\Delta \theta \leftarrow f_{out2};$ 12:  $\left[R_{x},R_{y},\theta_{R}]\leftarrow RobotState.update([step,\Delta \theta ]\right)$ 13:  $dis_{RG}.update([R_{x},R_{y}]);$ 14:  $Path.append([R_{x},R_{y}]);$ 15: end   While; 16: return   Path;

According to the algorithm flow, the information necessary for planning is entered before the beginning, including map data, starting and target state points plus information about the robot and the sensors it carries. *InitRobotState* is a function that initializes the robot states and returns the initial pose of the SMR, where $R_{x}$, $R_{y}$ are the horizontal and vertical coordinates of the robot and $\theta_{R}$ denotes the orientation (pose). When the distance to the target exceeds a preset threshold, the robot begins to continuously acquire the distribution of surrounding obstacles via its sensors. The function RangeSensor represents the sensor feedback function and returns the minimum distance $dis_{RO\min}$ between the robot and nearby obstacles, detected by all sensors, as well as the angle $\theta_{GRO}$ formed between the vector from the robot’s centroid to the nearest obstacle and the vector toward the target point. The robot is equipped with nine sensors oriented at angles of $-\frac{\pi }{3}$, $-\frac{\pi }{4}$, $-\frac{\pi }{6}$, $-\frac{\pi }{12}$, 0, $\frac{\pi }{12}$, $\frac{\pi }{6}$, $\frac{\pi }{4}$ and $\frac{\pi }{3}$ relative to the robot’s heading direction $\theta_{R}$. The principle of sensor-based data acquisition is illustrated in Figure 1. Before recording sensor information, the robot’s current position is recorded to initialize the Path.

**Figure 2:**
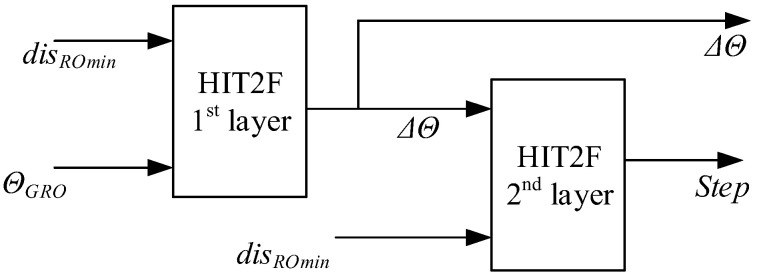
Hierarchical type-2 fuzzy inference system of GA-HIT2F.

The sensor data is fed into a hierarchical interval type-2 fuzzy logic system (IT2 FLS) with two inputs: $f_{in1}$ and $f_{in2}$. Here, $f_{in1}$ is the normalized value of the minimum obstacle distance $dis_{RO\min}$, scaled to match the expected input range, and $f_{in2}$ corresponds directly to the angle $\theta_{GRO}$. The system features a two-layer structure: the first layer outputs a steering adjustment Δθ, which is then combined with $dis_{RO\min}$ as input to the second layer for further fuzzy inference. The second layer finally produces the step size for the IMR. The overall input–output architecture of this GAHIT2Fsystem is illustrated in Figure 2. Once the outputs are obtained from the type-2 FIS, the step size is scaled by a map-dependent multiplier and then used together with Δθ to actuate the robot for the current motion step. The robot’s state is updated to record its new pose, which is appended to the planned path. Simultaneously, the target distance $dis_{RG}$ is recalculated. The path-planning loop terminates when the IMR enters a threshold region around the target, at which point the final path is output.

### 2.2. Principle of Parameter Optimization of the Affiliation Functions in GA-HIT2F

During the development of the GA-HIT2F system, the path planner employs a two-layer fuzzy rule design to enhance the robot’s obstacle avoidance performance. All variables utilize interval type-2 fuzzy sets, with both upper and lower membership functions represented by triangular membership curves. Within each layer, every input variable is divided into five linguistic variables across its respective universe of discourse. For definitions of the fuzzy sets associated with these linguistic variables and their corresponding membership functions, please refer to [22].

The initial parameters of the membership functions are set empirically. To further optimize the decision-making at each step of the path planning process, the system applies a Genetic Algorithm (GA) to globally optimize all parameters of the triangular membership functions. This optimization enables the Intelligent Mobile Robot (IMR) to execute more appropriate obstacle avoidance actions, namely step length and steering angle, based on its current state. The design of the fitness function used in the GA optimization is described as follows:

(1) $$fit_{HIT2F}\left(paras\right)=fit_{1}+fit_{2}+fit_{3}-n$$

where paras is the combination of all parameters of the triangular affiliation function, about 68 parameters in total, $fit_{1}$ is the length of the path obtained from one independent path planning, $fit_{2}$ indicates the distance between the intelligent body and the target at the termination of the current round of path planning, $fit_{3}$ indicates the penalty term of the feedback if the SMR still has not arrived or has moved away from the target area when the set maximum number of planning steps is reached, and n is a constant related to the map, robot, and sensor scale.

### 2.3. Parameter Optimization Performance and Its Defect Analysis

**Figure 3:**
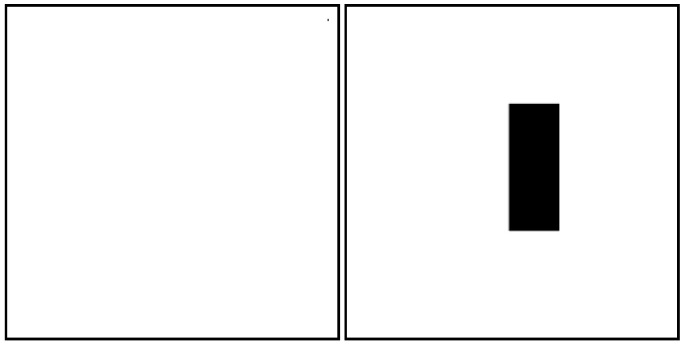
Empty environment (**left**) and central obstacle environment (**right**).

According to the fuzzy rules of GAHIT2Fsystem we can observe the general mechanism of GA-HIT2F path planner: when there is an obstacle around the robot, the robot is empowered with the steering angle of the tendency to deviate from the obstacle, and according to the distance of the nearest obstacle, the robot is assigned with the appropriate step to enable the robot to maintain better safety with the surrounding environment after moving forward. For example, when the intelligent body detects an obstacle within a short distance to the left of a large angle, according to rule 1, the LPP planner GA-HIT2F outputs a control amount of deflection to the right and advances at a smaller speed (step length) to avoid the risk of collision. We note that GA-HIT2F defaults to zero degrees when there is no obstacle directly in front of the robot, then according to fuzzy rules 2 to 5, the robot will execute the turning action, and obviously the decision is unwise and non-optimal. For this conservative and reckless collision avoidance behavior, this paper finds that GA-HIT2F will compensate for this strategy by optimizing the parameters through GA, i.e., the process of GA search for the optimal parameters will judge the robot’s tendency to approach the target as much as possible so that the robot will attempt to go straight to the target position if the safety is assured in front. However, this also generates a problem that the optimized parameters only fit the basic information set in the optimization process; therefore, when a new target or a new map emerged, the GA-optimized parameters will have a tendency to move conforming to the original map and the original target hastily, because the parameters are the optimal values found by the GA after a large number of iterations in the old one. The parameters are not suitable for planning in other targets and locations. In this section, a small planning test was conducted to clarify this phenomenon. Two types of maps are prepared as follows: the first is a blank environment without any obstacles, and the theoretical optimal path of the robot is the line between the starting and target points; the second is an environment with a rectangular obstacle in the center of the map, as shown in Figure 3.

**Figure 4:**
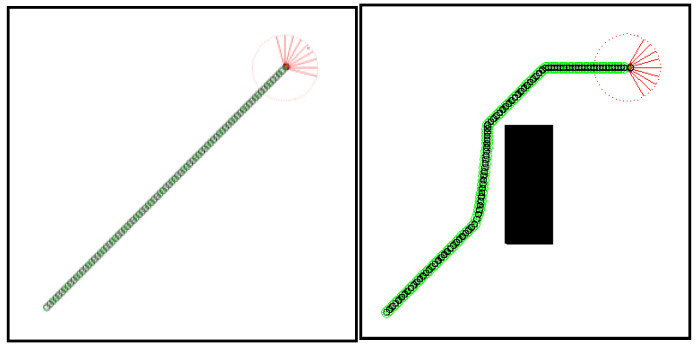
LPP results of GA-HIT2F for two environments.

The GA-HIT2F path planning (LPP) for the two environments is performed first, that is: after optimizing the optimal parameters based on the respective map and starting target information using GA, the parameters are input into the GAHIT2Fsystem and then the path planning is completed using Algorithm 1, and the results are shown in Figure 4.

In particular, in the empty environment, the optimal parameters successfully allow the robot to perform a direct action towards the target with a two-point line, which fully demonstrates that the GA-optimized parameters enable the robot to make fine adjustments to take optimal actions under the original fuzzy rules.

**Figure 5:**
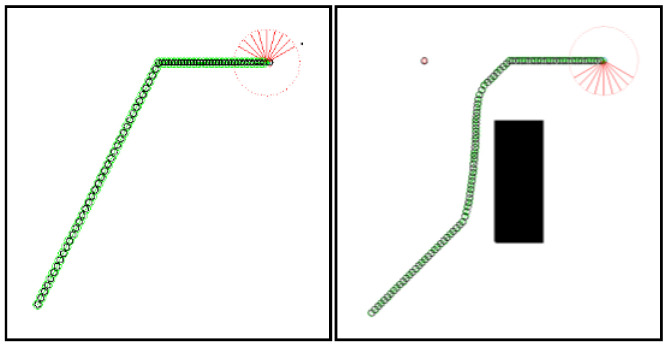
Results of GA-HIT2F for two environments with changed parameters.

To further explore the effects of the parameters, two special tests were conducted: (1) changing the parameters in the Figure 1 environment, i.e., using the optimized parameters of Figure 2 environment for the path planning of Figure 1; (2) changing the target points of the Figure 2 environment, but still taking the GA-optimized parameters in the original target state. The planning results obtained from the two tests are shown in Figure 4 and Figure 5.

From the results of test (1), it can be seen that the robot unexpectedly turns to avoid an “obstacle” in an otherwise empty environment, ignoring the original target point. These weird behaviors reinforce the idea in this section that the optimization parameters had already contained some key information about the original map, and even if they are applied to other maps, the robot would still make a decision to move towards the original map or the original target.

**Figure 6:**
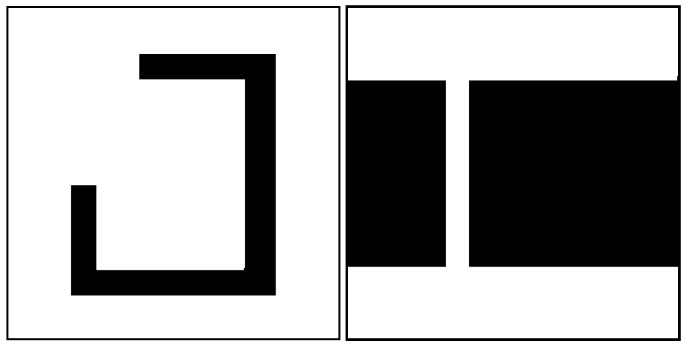
Special environments with concave surfaces (**left**) or narrow walls (**right**).

GA-HIT2F as an LPP planner possesses a defect that is basically difficult to be solved by a class of LPP algorithms. In a special environment such as the concave wall or the narrow passage wall in Figure 6, SMR often falls into local extremes, resulting in poor path planning. The GA-HIT2F algorithm causes this phenomenon because SMR first has to avoid the wall when traveling to the target point, and when it bypasses the wall, it should theoretically advance to the end point, but at this time, due to this special wall environment, it must cause the robot to produce the exact same fuzzy input at a certain location as before bypassing the wall, plus using the same optimization parameters. The two coincidences of sameness make the GAHIT2Fsystem produce a steering output command instead, causing the robot to move further away from the target.

To summarize, here are two major defects of the basic GA-HIT2F algorithm parameters: irst, if the map or target point changes, the fixed parameters of GA optimization must be re-optimized. Second, in some wall environments with local minimum traps, unexpectedly the same fuzzy inputs with fixed optimization parameters cause the robot, which should have taken two different strategies for obstacle avoidance, to produce the same fuzzy outputs to make the path planning fail.

### 2.4. The Necessity of Dynamically Adjusting the Parameters of the Affiliation Function

Recognizing the limitations of the parameter optimization approach in the basic GA-HIT2F, this paper argues that its fixed optimized parameters are obtained by seeking a balance between the robot and the overall map environment, including the start and end points. Consequently, this method struggles to effectively capture the distribution relationship among the three core elements: the robot, varying surrounding obstacles, and the target point. Ideally, fuzzy operations require parameters capable of reflecting the real-time state of the robot, encompassing sensor-detected obstacle information and the target point status. In other words, the parameters of the membership functions should be dynamically adjustable and possess the self-adaptive ability to select values that are optimal specifically for the current time step under each distinct configuration of the three elements.

## 3. QL-HIT2F Planner Based on Reinforcement Learning and the Concept of Average Obstacle Orientation

### 3.1. Average Obstacle Orientation

In the GA-HIT2F algorithm, sensors in nine directions are used to detect the distance to obstacles within their respective detection ranges. The *RangeSensor* function returns the nearest angle $\theta_{GRO}$, which is specifically obtained by subtracting $\theta_{GR}$ from $\theta_{RO}$. Here, $\theta_{GR}$ represents the angle of the vector from the IMR to the goal, while $\theta_{RO}$. represents the angle of the vector from the IMR to the nearest obstacle. $\theta_{RO}$ reflects, to some extent, the positional relationship between the agent and environmental threats, but it only considers the distribution of the nearest obstacle. This makes it difficult to reflect obstacle features collected by sensors in other directions, leaving information from the unknown environment in other directions underutilized within the HIT2F system. To comprehensively consider obstacle information from all directions, ideally, the closer an obstacle in a certain direction is to the robot, the greater its impact on the robot should be. Moreover, such influences from obstacles in all nine directions should be aggregated. Therefore, this paper proposes a new concept called the average obstacle orientation angle (AOO) [26], which is an average tendency angle calculated based on the distance and angle information of obstacles detected by each sensor, representing the overall distribution of obstacles around the robot. It is computed according to the following formula:

(2) $$\theta_{ARO}=\frac{\sum_{i=1}^{i=9}w_{i}\theta_{RO_{i}}}{\sum_{i=1}^{i=9}w_{i}}$$

where $w_{i}$ is the weight of each sensor corresponding to the obstacle and $w_{i}=1/dis_{ROi}$. So the function *RangeSensor*, which incorporates the concept of AOO, should return a value of $\theta_{GRO}$ as $\theta_{GRO}=\theta_{GR}-\theta_{ARO}$. However, a limitation of the average orientation angle is that when multiple independent obstacles are distributed on both sides of the robot, their positive and negative angles relative to the robot’s heading cancel each other out during the weighted averaging process. Consequently, a correction to the angle $\theta_{ARO}$ is required, as detailed below.

(3) $$\theta_{GRO}=\left\{\begin{array}{l} \theta_{GR}-\theta_{RO},\;\;\;\;\;\left|\theta_{ARO}-\theta_{RO}\right|>\theta_{thr}\\ \theta_{GR}-\theta_{ARO},\;\;\;\;\;others \end{array}\right.$$

where $\theta_{thr}$ is the threshold value for the difference between the AOO and the orientation of the nearest obstacle. The implication of this formula is that when independent obstacles exist on both sides of the robot, $\theta_{ARO}$ may deviate excessively, rendering the estimation based on the average orientation inaccurate. In such cases, the AOO angle is not applied.

### 3.2. Dynamic Parameter Configuration of Reinforcement Learning

Q-Learning algorithm [24,25,26] (QL) is a category of discrete reinforcement learning methods based on Q-tables, which implements unknown environment exploration and action mapping based on a reward and punishment mechanism. QL is generally employed in path planning in two ways. The first is employing it as a main method to train a mobile robot to seek an optimized path through corresponding state-action policy; the second approach is employing QL as a policy maker of some parameters or obstacle avoidance strategy to support path planning methods through its learning process; for instance, studies [24,25] provide explanatory examples, respectively. QL can be understood in the field of SMR path planning as follows: the SMR chooses an appropriate obstacle avoidance strategy in the current state based on Q-tables using some existing experience and executes it. The robot is driven to navigate its environment and conversely, the environment provides timely feedback on the reward; meanwhile, the SMR arrives at the next state. This executed strategy is interpreted as an action of the state transition. In addition, the reward and the new state will successively update the Q table using the Bellman equation (Algorithm 2) and then backfire on the robot to prompt it to dynamically adjust its actions so that it can continuously learn through trial and error to achieve the optimal policy. The standard QL reinforcement learning algorithm is shown as follows.
**Algorithm 2** Standard Q-Learning algorithm1: $Q_{\mathrm{Table}}\leftarrow \mathrm{Initializezeros}(NumS,NumA)$ 2: **for** preset episodes 3:  $S_{t}\leftarrow \mathrm{InitRandomState}(S)$ 4: **While** $S_{t}\neq terminal\;state$ 5:  $A_{t}\leftarrow \mathrm{GreedyChooseAction}\left(S_{t},Q_{\mathrm{Table}}\right)$ 6:  $S_{t+1},R\leftarrow \mathrm{PerformActionToGetEnvFeedback}\left(S_{t},A_{t}\right)$ 7:  $Q\left(S_{t},A_{t}\right)=Q\left(S_{t},A_{t}\right)+\alpha \left(R+\gamma \cdot \max Q\left(S_{t+1},A_{t}\right)-Q\left(S_{t},A_{t}\right)\right)$ 8:  $S_{t}\leftarrow S_{t+1}$ 9:    **end while** 10:   **end for**

Due to the complex unknown environment of path planning in which the possibility of obstacles with special shapes emerging is high, it is necessary to change them dynamically according to the robot’s state. Considering the extreme environment of narrow passages as in Figure 6, it is found through testing that the GA-HIT2F algorithm does not easily enable the robot to traverse narrow areas. After profound investigation, we find that this dilemma is caused by the fact that the maximum radius of robot detection $dis_{range}$ (which is also the minimum safe distance of SMR) is too big relative to the width of the narrow passage. When the robot travels near the narrow area, it detects an obstacle within $dis_{range}$. Since $dis_{range}$ is greater than the narrow passage width, the robot misjudges that the detection range is full of obstacles, leading to avoidance of the surrounding narrow passage entrance. Given that the maximum detection range of the robot sensor itself is fixed, we propose to adaptably change the value of $dis_{range}$ involved in the operation in real time in the *RangeSensor* function instead of using a fixed one of the sensor itself; i.e., $dis_{range}$ is also considered as a unfixed parameter that needs to be dynamically adjusted by QL, denoted by $dis_{rangeincoding}$ ($dis_{rangeincoding}\leq dis_{range}$).

Additionally, this paper introduces the concept of the average obstacle orientation angle $\theta_{ARO}$ and specifies a threshold condition for its activation. However, since the threshold $\;\theta_{thr}$ is manually set, relying solely on this criterion to decide whether to activate $\theta_{ARO}$ may not be sufficiently robust across different specialized environments. To address this, an activation switch $Switch_{ARO}$ is further proposed. This switch is a binary logic variable that can only take the values 0 or 1, with $\theta_{ARO}$ being activated only when $Switch_{ARO}$ is turned on (set to 1). In summary, $Switch_{ARO}$ is also a parameter that should be dynamically adjusted through Q-learning decision-making. Therefore, the average obstacle orientation angle will be activated only when the following condition is satisfied:

(4) $$\;\left|\theta_{ARO}-\theta_{RO}\right|>\theta_{thr}\;\;\;\&\&\;\;\;Switch_{ARO}=1$$

The parameters that need to be adjusted dynamically using QL in The QL-HIT2F algorithm in the process of LPP in unknown environment are: the parameters of all affiliation functions in the fuzzy system GAHIT2F, the value of detection radius $dis_{rangeincoding}$ in the function RangeSensor and the AOO activation switch $Switch_{ARO}$, totaling 70 dynamic parameters.

### 3.3. Reinforcement Learning State-Space for QL-HIT2F Path Planner

In the QL-HIT2F path planning algorithm, considering that reinforcement learning requires generalization to different maps and environments, the relative relationships among the trinity (the robot state, the surrounding obstacle information detected by the sensors, and the target point) should be utilized to constitute the state space of QL (S), which together form a specific state uniquely describing the current features at a given moment. These relative relations are determined in this paper by choosing the following defined four variables (1) the Euclidean distance $dis_{RG}$ between the current position of the robot and the target; (2) the angle $\theta_{RRG}$ between the current robot orientation and the line connected between the robot and the target point; (3) the orientation angle $\theta_{RO}$ of the nearest obstacle detected by the sensors; and (4) the steering angle Δθ of the outputs of the QL-HIT2F system at current control time step. The values of the above four variables determine the four states st1, st2, st3 and st4. The first two states measure the extent to which the intelligent agent is close to or far from the target. The third reacts to the distribution of obstacles, and the last state indicates the intensity of the steering control of the current planner; if the Δθ is greater, it implies that the planner had made some kind of dangerous judgment with giving the robot a greater degree of deviation, which indirectly reflects the possibility of collision occurrence in the surrounding environment and safety level of it. The numbered discrete values of each state are listed in Table 1, which shows that the four small states define a total of $C_{2}^{1}C_{3}^{1}C_{4}^{1}C_{2}^{1}=48$ 48-dimensional state space S; here, the threshold values of these variables are selected similarly to [26].

The definition of what kind of action to perform in a particular state and what level of reward the SMR obtains after transferring into a new state in the next step through action selection can be the key issue for the QL algorithm to train the agent for policy making. The planner generates control commanders by means of two sets of fuzzy rules formulated in the GAHIT2Fsystem and dynamic QL optimized parameters. According to the analysis in the previous sections, the optimization parameters that require QL to achieve dynamic self-adaptation can be divided into two groups, the affiliation-parameter group and the other-variable parameter group. The fuzzy input $dis_{RO\min}$ in the first group corresponds to 26 parameters, $\theta_{GRO}$ corresponds to 21 parameters, and input Δθ corresponds to 21 parameters; the total of about 68 parameters of this group is now unified into one vector parameter paras. The other-variable parameter group have the radius $dis_{rangeincoding}$ and the AOO activation switch $Switch_{ARO}$. The parameters that constitute the QL action space are thus reduced to three large variables. To sum up, the action space can be generated by combinations of the possible values of each of these three parameters. The design and definition of the QL action space are elaborated in the following sections.

### 3.4. Reinforcement Learning Action Space for QL-HIT2F Path Planner

**Figure 7:**
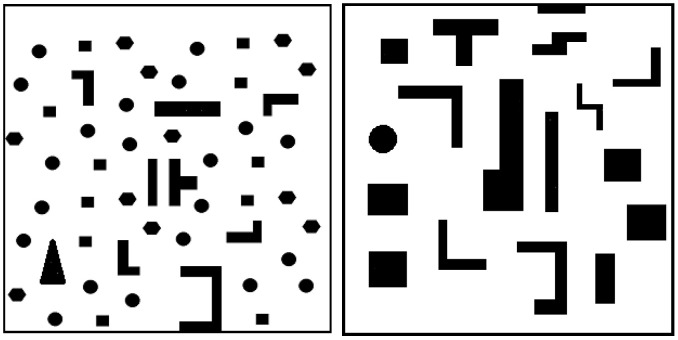
Meta-map 1 and meta-map 2 for two environments.

The Q-Learning algorithm records all the discrete state-action pairs defined in the Q-Learning algorithm, and the values of the dynamic parameters should be determined carefully in order to reduce the complexity of the action space. A new concept, the meta-map, is introduced before discussing the values of the optimization parameters. Since the initial intention of employing reinforcement learning and QL is to make the parameter values dynamically change and automatically adapt to different environment maps, the unknown environment used in training the agent (the robot) for QL to complete path planning must be a map specifically designed to contain various special obstacles. Once the robot has explored the environment and learned the characteristics of the obstacles by trial and error, it can make optimal decisions in any other maps without having to separately solve the optimization parameters of the corresponding maps. In this paper, we refer to the map used by QL for training as a ‘meta-map’, as shown in Figure 7, where we specifically designed the meta-map containing narrow passages and concave walls, as well as other types of multi-state obstacles.

**Figure 8:**
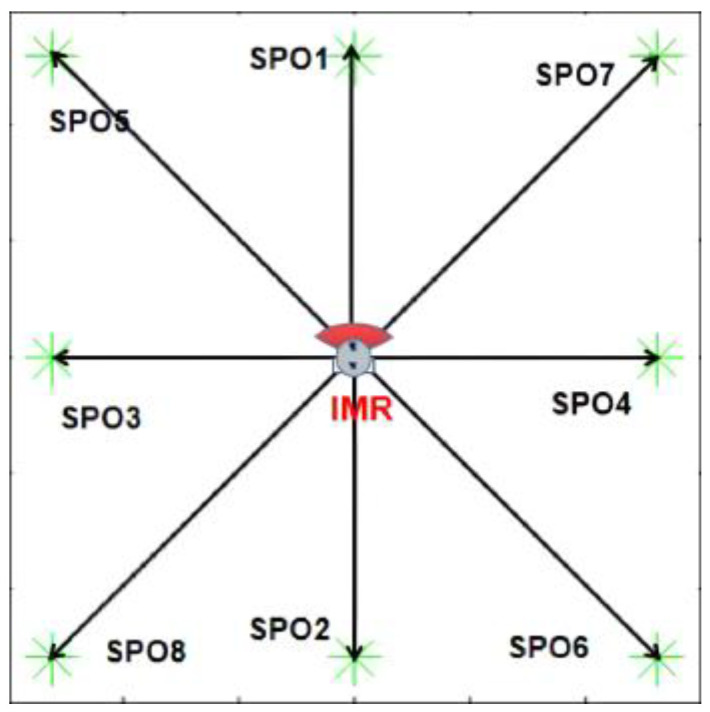
Schematic diagram of the eight directions of SPO.

The previous subsection has clarified that the parameters obtained from the original GA-HIT2F optimization can only adapt to the same environment and the same target point. Even if the target point is changed, the robot will tend to converge to the original target. Based on this, this paper solves this shortcoming by optimizing the parameters independently with multiple fixed targets, which means this paper does not adopt the target of path planning to optimize the parameters, but selects eight special points to optimize the parameters eight times, and the eight optimized parameters represent, respectively, eight kinds of tendencies to move (to the front, back, left, right, left oblique, and right oblique). In order to get the parameters that can endow the SMR with eight types of movement tendencies, considering that the LPP is in an unknown environment where the a priori information about obstacles cannot be obtained, In this paper, we extract eight SPOs, (Subgoal for Parameters Optimization) on the map boundary as shown in Figure 8 below, which correspond to eight types of tendencies of the robot movement, and these eight SPOs are applied to optimize the affiliation parameters of these eight extreme scenarios in meta-maps independently using the original GA-HIT2F system. (Because we only need the moving tendencies in each direction, GA-HIT2F itself does not have special complex environment planning capability but only uses GA to obtain the optimal parameters for each tendency, and the ability to traverse extreme environments requires QL training in the meta-maps). That means paras, the dynamic parameters in the QL spaces, possesses eight possible values, which can be expressed as the following equation.

***paras*** ∈ {***pa1***, ***pa2***, ***pa3***, ***pa4***, ***pa5***, ***pa6***, ***pa7***, ***pa8***} (5)

where pak denotes the SPOk of the kth direction in the simple meta-map, and they are all vectors consisting of about 70 affiliation parameters.

The set of values of other affiliation functions-related parameters are given below, and the discrete range of the radius $dis_{rangeincoding}$ of the SMR in the sensor operation process is defined as follows: $dis_{range\_in\_coding}\in \left\{\frac{1}{3}dis_{range},\;\;\;\frac{2}{3}dis_{range},\;\;\;dis_{range}\right\}$.

The binary switch variable $Switch_{ARO}$ that activates the AOO is expressed by a binary logic, which takes the following values, i.e., $Switch_{ARO}\in \left\{0,1\right\}$.

In summary, according to the multiplicative principle, there are a total of $C_{8}^{1}C_{3}^{1}C_{2}^{1}=48$ possible combinations for the two groups of parameters that constitute the QL action space, i.e., an action space with 48 dimensions is defined. A certain action representation of the system is like: $paras=pa_{1}$ and $dis_{rangeincoding}=\frac{1}{3}dis_{range}$, and $Switch_{ARO}=0$.

Specifically, the above described SMR states and action space generate a 48×48 dimensional matrix $Q_{Table}$. The principles of environmental reward and the whole procedure of QL training the agent will be discussed in detail in the subsequent sections.

### 3.5. Reinforcement Learning Reward Mechanism and Training Process for QL-HIT2F Path Planner

A reasonable reward mechanism will make an accurate performance assessment of each step executed by the agent in path planning, and the continuously accumulated rewards or penalties for a specific state indirectly assess the current degree of safety of the robot with respect to its surrounding environment. In this paper, the reward principle is set up as follows in Table 2, and the reward score is determined by the values of four new defined states formed after performing a specific action. These four states are: the distance between the robot and the target, the proximity to the target, the length of the feasible path when successful arrival is satisfied, and last the robot’s position.

To achieve a perfect post-training intelligence, the design of the policy for choosing one step from the action space in each training step of the QL algorithm is also crucial. The greedy strategy avoids pure exploration or unilateral exploitation, and it is often used as a reference for action selection in the QL training. However, in the unknown environment, all states from the state space generated by the agent are discrete, and if this state has not shifted compared to the previous step, no action should be generated according to the greedy strategy. Assuming the second case, in the early episodes at the beginning of training, $Q_{Table}$ is not updated completely and a discrete state of the robot will correspond to multiple discrete actions with an equal value (all are 0); this situation also cannot retain the greedy strategy, but continues the discrete actions of the previous step to maintain the continuity of robot control. In summary, the QL training process in this paper is shown in Algorithms 3 and 4.
**Algorithm 3** QL training flow for QL-HIT2F path planner1: Input necessary Map and Robot preliminary information 2: $S\leftarrow \mathrm{InitQLStateSpac}e\left(\right);$ 3: $A\leftarrow \mathrm{InitQLActionSpace}\left(\right);$ 4: $Q_{Table}\leftarrow \mathrm{InitializeZeros}\left(48,48\right);$ 5: for preset  episodes do 6:  $st1,st2,st3,st4\leftarrow \mathrm{InitRobotState}\left(\right);$ 7:  $S_{t}\leftarrow \mathrm{QStatesMapping}\left(st1,st2,st3,st4\right);$ 8:  $while\;\;\;S_{t}\neq terminal\;state$ 9:    $\begin{array}{l} A_{t}\leftarrow \mathrm{ModifiedGreedyChooseAction}\\ \left(S_{t},\;A_{t}(last),Q_{Table},StaticFlag,\;\right); \end{array}$ 10:  $\begin{array}{l} {}[paras,dis_{RangeInCoding},Switch_{ARO}]=\\ \mathrm{QActionDemapping}\left(A,A_{t}\right); \end{array}$ 11:  $RobotState.update\leftarrow \mathrm{HIT}2F\left(3\;\;parameters\right);$ 12:  st1,st2,st3,st4←RobotState(new); 13:  $S_{t+1}\leftarrow \mathrm{QStatesMapping}\left(st1,st2,st3,st4\right);$ 14:  $\begin{array}{l} {}[R,StopFlag]\leftarrow \mathrm{PerformActToGetEnvFeedBack}\\ \left(RobotState(new)\right); \end{array}$ 15:  $Q_{Table}.update(S_{t},S_{t+1},A_{t},R);$ 16:   $S_{t}(last)=S_{t};$ 17:   $S_{t}=S_{t+1};$ 18:   StaticFlag=0; 19:   $if\;S_{t}=S_{t}(last),\;StaticFlag=1;end\;if$ 20:   if StopFlag=1, break;end if 21:    end while 22: end for 23:    $\mathrm{return}\;Q_{Table}$

In this algorithm, InitQLStateSpace(), and InitQLActionSpace() initialize the state space and action space in reinforcement learning separately. RobotState(new) means the new updated state of the robot. InitializeZeros() initializes a Q-value table with all values set to 0. InitRobotState() initializes the actual physical state of the robot at the beginning of a new turn, which returns four state variables (st1, st2, st3, st4), which are raw sensor or environmental data. QStatesMapping() maps the current actual physical state of the robot to the discrete state index used in the Q table. ModifiedGreedyChooseAction() selects action $A_{t}$ based on the current state $s_{t}$, previous action $A_{t}$ (last), Q-table, and *StaticFlag*. QActionDemapping() decodes the abstract action index $A_{t}$ selected from the Q table into actual parameters that can directly control the HIT2F planner. HIT2F(), means that the function executes the core path planning algorithm (HIT2F) proposed in this study, it receives parameters obtained from the previous QActionDemapping() step, calculates the robot’s next motion command, and updates its physical state accordingly. RobotState(new) means the new updated state of the robot. PerformActToGetEnvFeedBack() evaluates the new state and returns an immediate reward R and a termination flag *StopFlag*.

QL training generally consists of two cycles: the outer cycle is the number of reinforcement learning episodes, according to experience set to 5000–20,000 times; the inner cycle represents an independent path planning process, in which the loop may be interrupted because the agent has reached the target or collision occurs with the environment. The *QStatesMapping* function in the pseudocode represents the state mapping function, which maps to a specific state in the state space based on the four predefined decision variables of the current robot. The numbered $S_{t}$ reflects the specific state that the robot occupies in the discrete state space at this moment. Inversely, the *QActionDemapping* in the algorithm will absorb the specific actions represented by discrete number $A_{t}$ from the modified version of the greedy policy (detailed selection policy is as represented in Algorithm 4) and then demap the action into values of specific dynamic parameters. Once the current parameter values are determined, they can be integrated into the HIT2F system for path planning. The robot then moves one step to the new state through this planner, and after the state is updated, the *PerformActToGetEnvFeedBack* function is immediately triggered to give the robot the reward or punishment feedback it should obtain, after which Q can be updated based on the triplet (state, action, and reward) using the Bellman equation.

There are a few details in the algorithm that are worth noting. The variable StopFlag represents the flag that compels the robot to terminate the training of a single episode after a collision interaction with an obstacle, and StaticFlag represents the flag that the robot is supposed to keep the previous action group; both flags work when they are set to 1. In addition, the policy of action selection of QL considered the continuous change in robot actions; the *CoverZeros* function in Algorithm 4 represents that all the rows corresponding to a state in the Q table are 0, when the actions should also be generated completely randomly.
**Algorithm 4** Modified greedy choose action algorithm1:  if  StaticFlag=1 2:  $A_{t}=A_{t}\left(last\right);$ 3: $\mathrm{elif}\;\;rand\leq \varepsilon \;\;\;or\;\;\;\;\mathrm{CoverZeros}\left(Q_{Table}\left(S_{t}\right)\right)$ 4:  $A_{t}\leftarrow \mathrm{RandAction}\left(A\right);$ 5: else  6:  $A_{temp}\leftarrow \underset{A_{temp}\in A}{\mathrm{argmax}}\left(Q_{Table}\left(S_{t}\right)\right);$ 7:   $\mathrm{if}\;\;\mathrm{length}\left(A_{temp}\right)\;>1\;\;$ 8:   $A_{t}\leftarrow A_{temp}\left(rand\right);$ 9:   else 10:    $A_{t}=A_{temp};$ 11:  end if 12: end  if 13: $\mathrm{return}\;A_{t}$

## 4. QL-HIT2F Path Planning Training and Simulation Test

### 4.1. Path Planning Training of QL-HIT2F for Metamap Scenarios

This study adopts a sequential training paradigm to learn a versatile Q table that empowers the IMR to process a wide range of unknown obstacles in real time across unfamiliar maps. Using the QL-HIT2F algorithm, training is conducted successively on two distinct prototype maps. The process begins with a simpler map to derive an initial Q table which then serves as the foundation for subsequent training on a more complex map characterized by narrow passages. This sequential strategy is designed to boost the robot’s generalization capacity, ensuring comprehensive learning of the diverse structural configurations. Prototype map Figure 7(left) represents a dense-sparse environment, while prototype map Figure 7(right) is a complex environment filled with various special obstacles, predominantly narrow channels. These two environments encourage the IMR to autonomously explore and learn a rich set of state-action pairs.

For the robot and QL-related parameter settings for path planning training in the meta-map scenario, the map boundary length is 300 units, while the starting and target locations of the SMR are the lower left and upper right corners of the map at a distance of the SMR safety radius to the boundary, respectively, so that the robot can traverse as many special obstacle bodies and walls as possible from both sides of the diagonal line. The reinforcement learning episodes are 20,000 times, and QL updates the parameter A, in the equation. To further strengthen the generalization ability of the training results to adapt to the new environment, the training procedure is again enriched in this paper. The approach is to add additional training of path planning with symmetry in five directions at other points selected at the map boundary in addition to the learning of lower-left-upper-right route navigation planning in QL training of each meta-map.

**Figure 9:**
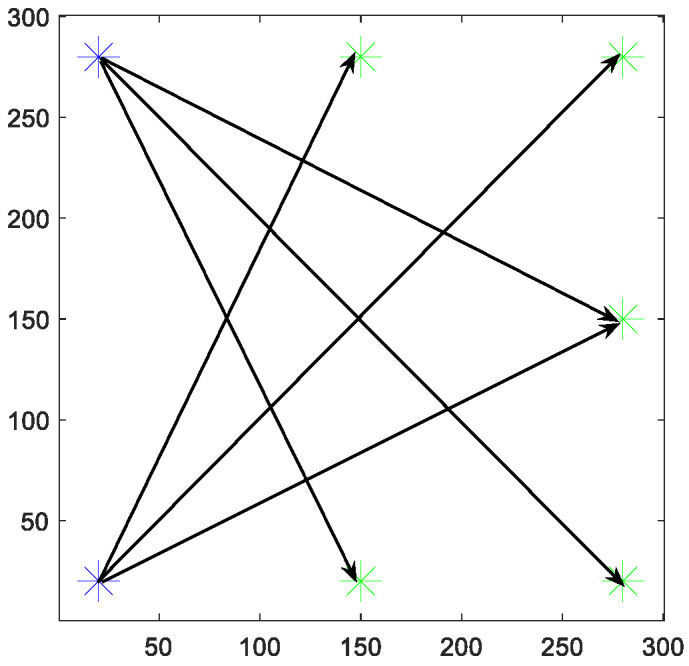
Schematic diagram of sub-training. Blue star is the starting point, and the green one represents the ending point.

In conclusion, one complete QL training for each meta-map contains six sub-trainings in these six directions as in Table 3 and Figure 8 above. Similarly, the QT outputs of the first sub-training will be used as the initial Q table for the second sub-training, and the third, fourth, and fifth are repeated in the same way. Thus, after the sixth sub-training is finished, the QT is a rich value of the robot explored and updated from multiple directions after accumulation of obstacle-avoidance experience in six times of sub-trainings. The results of all QL trainings are shown in Figure 9. It can be seen that the robot has fully explored the environment of the meta-map from all directions and multiple possibilities after up to 20,000 episodes of learning to find feasible paths for different homotopic classes.

### 4.2. QL-HIT2F Algorithm Path Planning Test in Multiple Scenarios

**Figure 10:**
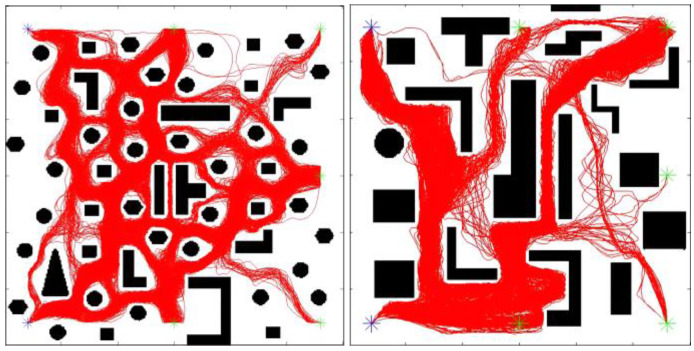
Q-Learning training results in meta-maps. The blue star is the starting point, and the green one represents the ending point.

**Figure 11:**
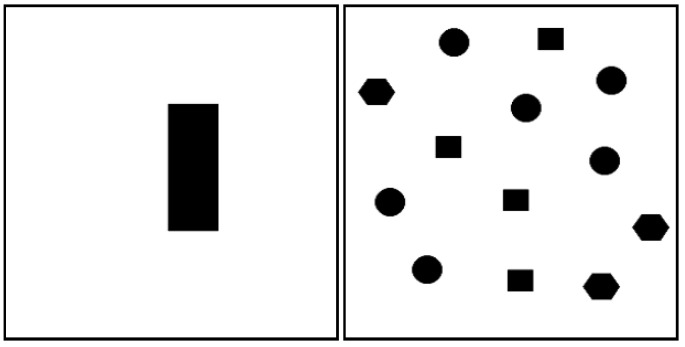
Basic scenarios.

**Figure 12:**
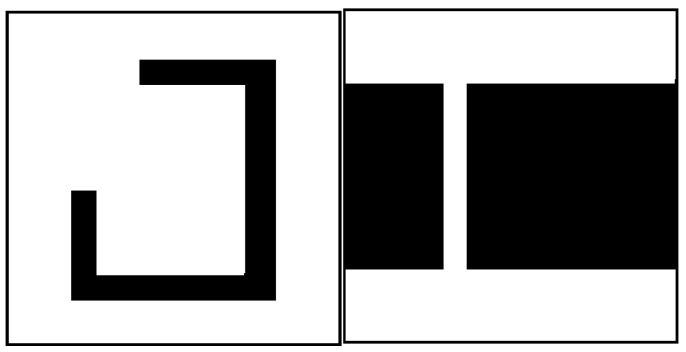
Special scenarios.

After sufficient training in the two meta-maps, the $Q_{Table}$ obtained by QL has contained the ability of how to dynamically adjust the parameters in the QL-HIT2F system when dealing with unknown environments, and SMR can then plan by reference to the $Q_{Table}$ to make the optimal parameter selection action. In this section, two scenarios (map1 and map2 of Figure 10) are selected based on [22] and one additional scenario (map3 and map4 of Figure 11 and Figure 12) with concave walls or narrow walls is designed to compare the proposed algorithm QL-HIT2F with the original planner GA-HIT2F. It should be noted that when path planning is performed in a new map, the obstacle environment is unknown, and the only information available to the path planning procedure are the starting and target points, the map boundary, the real time data detected by the sensors and a well-trained $Q_{Table}$. SMR needs to merely consume this information to complete the planning and navigation. The planning process of QL-HIT2F is basically the same as the original GA-HIT2F Algorithm 1 and the difference is the addition of two extra processes: one is the decision of the dynamic affiliation parameter based on the trained Q Table (how the determination is made is given in Training Algorithm 3) and the other is determination of whether to enable the AOO to calculate angles associated with the obstacle based on the AOO switch.

### 4.3. Path Planning Analysis in General Scenarios

**Figure 13:**
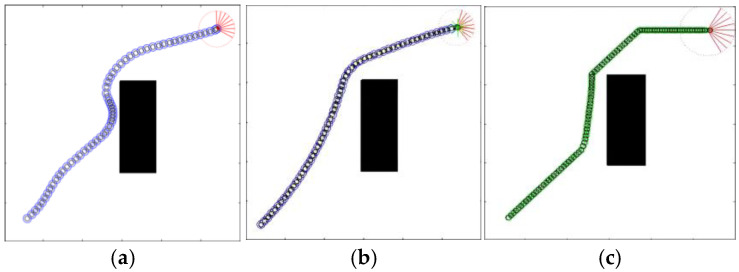
QL-HIT2F planning results without AOO (**a**), QL-HIT2F planning path with AOO (**b**) and GA-HIT2F planning path (**c**). The red part represents the ending point of a path, and the green star is the start point.

Initially, the LPP navigation test was conducted on the two maps used in the original literature, with the proposed QL-HIT2F algorithm benchmarked against GA-HIT2F. Both algorithms started navigation from identical initial positions and orientations. The path planning results are presented in Figure 13, where the actual robot paths are represented by blue-black circles for QL-HIT2F and green-black circles for GA-HIT2F.

Based on the two path planning results, it is clear that the LPP path for QL-HIT2F navigation outperforms GA-HIT2F in both the path length cost and the smoothness of the steering angle. The ability to flexibly judge collision hazards of SMR endowed by the training of reinforcement learning can be observed from the blue path. Although both paths successfully avoid the obstacle, the robot from the QL-HIT2F path starts to deflect slightly in advance of the green-black one from GA-HIT2F, and these slight turns cumulatively enable the robot to output the behavior of avoiding the central obstacle at an almost optimal position (near the upper left vertex of the obstacle’s convex hull), while for the green path, a sharper turn is made only at a moment very close to the surface of the obstacle and then robot’s obstacle avoidance is executed along the surface of the central obstacle. Moreover, after the robot bypassed the central obstacle, the GA-HIT2 algorithm made a more conservative action output in the unknown environment, and the robot reached the target point from the map boundary with an almost 45-degree attitude as an arc-shaped trajectory. However, the QL-HIT2F algorithm directly makes the optimal action to drive the robot directly forward to the target. On the other hand, as evidenced by Figure 13a,b, one can see that the introduction of the AOO mechanism results in a significantly smoother and safer planned path, successfully avoiding obstacles in both scenarios.

**Figure 14:**
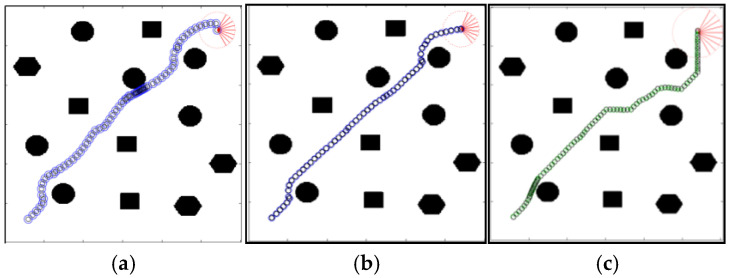
QL-HIT2F planning path without AOO (**a**), QL-HIT2F planning path with AOO (**b**) and GA-HIT2F planning path (**c**). The red part represents the ending point of a path.

In addition, the literature which proposed the GA-HIT2F planner also demonstrates the obstacle avoidance LPP navigation in dense multi-obstacle environments. Figure 14 below shows the comparison of the path planning results of our local planner QL-HIT2F with the original algorithm GA-HIT2F in such a multi-convex distributed map, from which more advantages of our QL-HIT2F can also be obviously perceived.

The QL-HIT2F planner demonstrates superior performance in Figure 14. Its path (Figure 14b) utilizes the Q-table to optimize dynamic parameters, resulting in a smoother, more continuous trajectory that avoids unnecessary turns and shortens the travel distance. This stands in sharp contrast to the GA-HIT2F path (Figure 14c), which is characterized by multiple turns and concludes with a redundant and unsafe near-90-degree reorientation of the robot immediately prior to reaching the goal. The results in Figure 14a,b also implies the effectiveness of the AOO mechanism.

**Figure 15:**
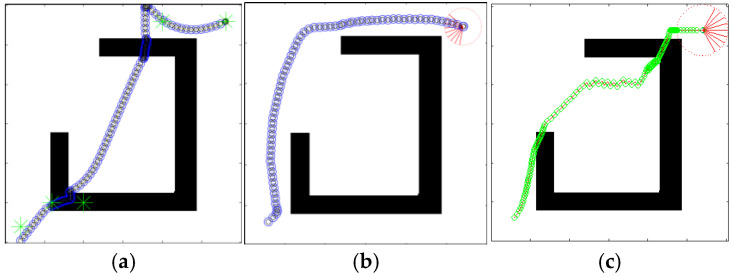
Planning results in concave scenario (QL-HIT2F without AOO (**a**), QL-HIT2F with AOO (**b**), GA-HIT2F (**c**)). Red part represents the ending point of a path, and the green star is the start point.

**Figure 16:**
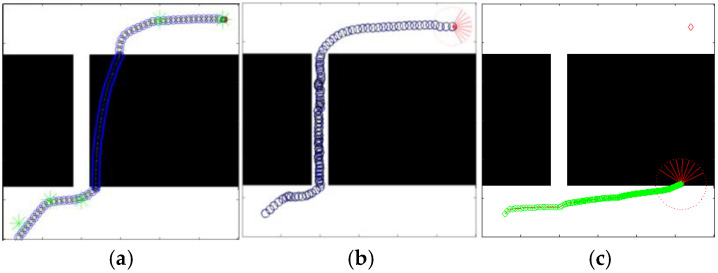
Planning results in narrow scenario 1 (QL-HIT2F without AOO (**a**), QL-HIT2F with AOO (**b**), GA-HIT2F (**c**)). Red part represents the ending point of a path, and the green star is the start point.

**Figure 17:**
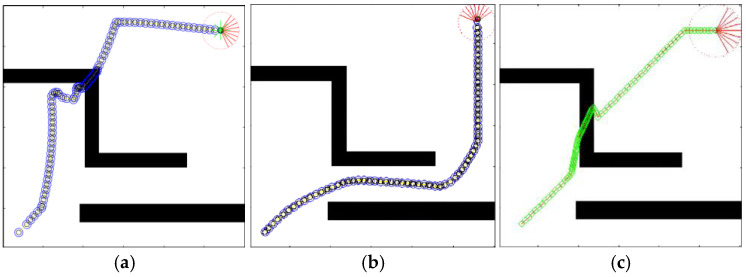
Planning results in narrow scenario 2 (QL-HIT2F without AOO (**a**), QL-HIT2F with AOO (**b**), GA-HIT2F (**c**)). Red part represents the ending point of a path.

In order to verify that the defects of the original algorithm elaborated in this paper can be effectively solved in QL-HIT2F, this paper additionally adds path planning with special obstacle walls, noting that the path planning is completely unknown and the robot does not foresee the special geometric patterns of the obstacles. The comparative results of the two algorithms are shown in Figure 15, Figure 16 and Figure 17. The comparative results demonstrate that the path planned by our algorithm (Figure 15b, Figure 16b and Figure 17b) successfully navigates past obstacles—including both horizontal narrow walls and concave walls—to reach the target. Notably, this is achieved without prior map knowledge, relying solely on real-time planning during motion. This performance confirms that the Q-table effectively selects optimal parameters and triggers the AOO mechanism at critical positions to escape local minima. In addition, the proposed method without AOO shows poor planning results, as listed in Figure 15a, Figure 16a and Figure 17a, the absence of the AOO mechanism led to a trajectory that collided with an obstacle, indicating a failure in path planning. Moreover, the original GA-HIT2 algorithm (Figure 15c, Figure 16c and Figure 17c) also fails as its path collides with obstacles and becomes trapped in a local minimum. This result directly corresponds to the limitation of the GA-HIT2F algorithm identified previously. In wall environments featuring local extremum traps, the algorithm’s fixed optimization parameters cause it to generate identical, inappropriate fuzzy outputs from similar inputs, even when a detour is essential. This inflexibility prevents the robot from adopting an obstacle-avoidance strategy, resulting in planning failure. These observations collectively verify that the QL-HIT2F algorithm successfully overcomes the limitations of its predecessor. It is capable of selecting optimal parameters in unknown and complex environments, making judicious collision-avoidance decisions to ensure successful navigation even in the presence of challenging concave walls.

The detail evaluations of the GA-HIT2F and QL-HIT2F for Figure 15, Figure 16 and Figure 17 are listed in Table 4; in this table, the Cost means the length of planning trajectory, and maxΔ*θ* denotes the maximum turning angle of planned path. Here, the results without AOO have also been expressed. Analysis of the statistical data in Table 4 reveals that the QL-HIT2F algorithm holds strong advantages not only in path cost but also in the steering angle (SA) variation during planning. Its maximum absolute SA variation remains within the robot’s ideal angular range, indicating superior motion continuity compared to the original GA-HIT2F. These results demonstrate that QL-HIT2F overcomes the limitations of the original algorithm by enabling optimal parameter selection in unknown and complex environments. Even in narrow areas with concave walls, it successfully guides the robot with reasonable collision-avoidance actions. Additionally, the data confirm that the introduced AOO mechanism effectively resolves collision avoidance, underscoring its critical role in the algorithm’s overall effectiveness.

## 5. Conclusions

This paper introduces QL-HIT2F, a dynamic adaptive local path-planning method that creatively combines Q-Learning to provide robots with decision-making autonomy for optimal action selection in unknown maps. The proposed approach features three major enhancements. First, a dynamic parameter adjustment in which each affiliation parameter can be adaptive to the changing triplet state; second, in addition to affiliation parameters in the original algorithm, a novel angle concept of obstacle orientation of average is introduced and the switch of whether it is activated or not is considered as an adaptive parameter; third, the operation value of the maximum radius of sensor perception of the robot is also considered as a dynamic parameter. In this study, Q-Learning is utilized to unify these three types of dynamic parameters for adaptive decision-making; based on this, the state space, action space and reward mechanism, three elements required for RL are designed on the basis of the mutual motion and geometric relationship among the triplet (the robot, the target, and the environment). The training empowers the robot with a universal policy to determine the optimal dynamic parameters which correspond to the current state of the robot in any unknown environment. Finally, a series of simulations are conducted, in general environments distributed with special obstacles and walls, respectively. The simulation results demonstrate that the actions and paths decided by the QL-HIT2F local planner outperform the original GA-HIT2F planner, which verifies the effectiveness of combining reinforcement learning with type-2 fuzzy dynamic tuning parameters to assist robot path planning in unknown environments.

## Figures and Tables

**Table 1:** Q-Learning state space.

State	Value	Dependence Variable
st1	1	$dis_{RG}<=dis_{start2goal}/2$
	2	$dis_{RG}>dis_{start2goal}/2$
st2	1	$0\leq \theta_{RRG}\leq 60{}^{\circ}$
	2	$-60{}^{\circ}\leq \theta_{RRG}<0$
	3	$\left|\theta_{RRG}\right|>60{}^{\circ}$
st3	1	$0<\theta_{RO}\leq 60{}^{\circ}$
	2	$-60{}^{\circ}\leq \theta_{RO}<0$
	3	$\left|\theta_{RO}\right|>60{}^{\circ}$
	4	$\theta_{RO}=0$
st4	1	$\left|\Delta \theta \right|\leq 60{}^{\circ}$
	2	$\left|\Delta \theta \right|>60{}^{\circ}$

**Table 2:** SMR’s environment reward mechanism and principles for QL training.

Variable	Reward Mechanism	Reward Principle
$dis_{RG}$	RW = RW + 5000	$dis_{RG}<=dis_{thr}$
$\Delta dis_{RG}$	RW = RW + 10	$\Delta dis_{RG}<0$
	RW = RW − 10	$\Delta dis_{RG}>0$
trajcost	RW = RW + 5000	trajcost(new)<trajcost(last)
$\left[R_{x},R_{y}\right]$	RW = RW − 200	Collision!

**Table 3:** Initial settings of QL sub-training for SMR.

	Start	Target	Initial
Sub-training1	$\left(20,20\right)$	$\left(280,280\right)$	45.00°
Sub-training2	$\left(20,20\right)$	$\left(280,150\right)$	26.57°
Sub-training3	$\left(20,20\right)$	$\left(150,280\right)$	63.43°
Sub-training4	$\left(20,280\right)$	$\left(280,20\right)$	−45.00°
Sub-training5	$\left(20,280\right)$	$\left(150,20\right)$	−63.43°
Sub-training6	$\left(20,280\right)$	$\left(280,150\right)$	−26.57°

**Table 4:** Evaluation of Path Planning Results for GA-HIT2F and QL-HIT2F.

Algorithm	Map	Cost	maxΔ*θ*	Steps
GA-HIT2F	map in Figure 13	384.79	43.21	99
	map in Figure 14	376.84	46.04	87
	maps in Figure 15, Figure 16 and Figure 17		planning failed	
QL-HIT2F (without AOO)	map in Figure 13	380.42	32.48	67
	map in Figure 14	368.37	28.41	62
	maps in Figure 15, Figure 16 and Figure 17		planning failed	
QL-HIT2F (with AOO)	map in Figure 13	377.26	17.80	60
	map in Figure 14	358.81	26.74	57
	map in Figure 15	305.46	36.43	47
	map in Figure 16	438.01	41.42	87
	map in Figure 17	415.75	24.29	64

## Data Availability

The datasets presented in this article are not readily available because the data are part of an ongoing study.

## Abbreviations

The following abbreviations are used in this manuscript:

GA-HIT2F	Genetic algorithm hierarchical interval type-2 fuzzy
QL-HIT2F	Q-Learning-supported adaptive hierarchical interval type-2 fuzzy
HIT2F	Hierarchical interval type-2 fuzzy
